# Selective Modulation of Lipid Langmuir Monolayers by Methyl Dehydrodieugenol: Insights into Its Interaction with Compressibility-Modulating Lipid Interfaces for Antiprotozoal Applications

**DOI:** 10.1021/acs.langmuir.5c02535

**Published:** 2025-07-14

**Authors:** Gabriel Torquato Berllini, Giulia Elisa G. Gonçalves, Felipe S. Sales, João Henrique G. Lago, Luciano Caseli

**Affiliations:** a Department of Chemistry, Federal University of São Paulo, Diadema, SP 09913-030, Brazil; b Center of Natural and Human Sciences, Federal University of ABC, Santo André, SP 09210-170, Brazil

## Abstract

This study investigates the interaction of methyl dehydrodieugenol (MDHDE) with model lipid monolayers to elucidate its potential as a membrane-targeting therapeutic agent. Langmuir monolayer experiments were conducted using dioleoylphosphatidylcholine (DOPC), dioleoylphosphatidylethanolamine (DOPE), and cholesterol to mimic the compressible and condensed states of protozoal and mammalian membranes. Surface pressure–area isotherms revealed that MDHDE reduced the molecular packing density of the lipids at biologically relevant surface pressures, suggesting attractive interactions at the molecular level. While the surface compressional modulus decreased for all monolayers, the dilatational elasticity measurements indicated an increase in the elastic character of the DOPC monolayer upon MDHDE incorporation. Surface potential measurements indicated distinct effects on electrostatic properties, with MDHDE causing a sustained decrease in potential for DOPC and convergence of values at collapse for DOPE. Brewster angle microscopy showed increased domain density in DOPC and coalescence of domains in DOPE upon MDHDE incorporation, whereas cholesterol monolayers were unaffected. Polarization-modulation infrared reflection-absorption spectra confirmed MDHDE-induced changes in lipid surface arrangement and hydration in both hydrophobic and hydrophilic regions of the monolayers, demonstrating selective interactions based on lipid composition. These findings highlight the MDHDE's ability to modulate membrane properties in a lipid-dependent manner, underscore the importance of lipid selectivity in designing membrane-active compounds, and provide a foundation for the further development of MDHDE as an antiprotozoal agent.

## Graphical abstract

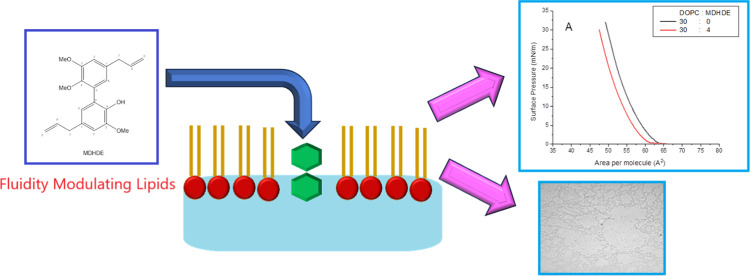

## 1 Introduction

Neglected tropical diseases (NTDs) encompass a group of infections that collectively affect nearly one billion people worldwide, particularly in regions with significant social and economic vulnerability. Among these, Chagas disease warrants special attention due to its substantial impact across Latin America.^1^ This disease is caused by (T. cruzi), a flagellate protozoan primarily transmitted to mammalian hosts by hematophagous triatomine bugs.^2^ Current treatments, including benznidazole and nifurtimox, are limited by reduced efficacy in the chronic stage and significant toxicity.^2–7^ The scarcity of effective therapeutic options, compounded by the absence of a vaccine, underscores the urgent need for novel drug discoveries. As a result, natural products represent a promising and underexplored resource in this context.^8,9^

Previous research has demonstrated the anti- activity of dehydrodieugenol (DHDE), methyl dehydrodieugenol (MDHDE), and dimethyl dehydrodieugenol (DMDHDE).^10,11^ These compounds showed activity against amastigote forms of *T. cruzi* with MDHDE displaying an IC_50_ of 8.2 μM, comparable to the positive control benznidazole (IC_50_ = 5.5 μM), while DHDE exhibited moderate potency (IC_50_ = 15.4 μM) and DMDHDE was inactive (IC_50_ > 100 μM).^11^ Building on these findings, this study evaluates the molecular interactions of methyl dehydrodieugenol (MDHDE) with membrane models using Langmuir phospholipid monolayers, a robust and widely used methodology for studying the interaction of bioactive compounds with lipid interfaces.^12–16^

The lipid models used in this study—DOPC, DOPE, and cholesterol—were carefully selected as single unsaturated components to serve as a continuation of a foundational step in a bottom-up approach to mimicking the complexity of protozoal and mammalian cell membranes.^11^ This strategy enables a controlled and systematic investigation of MDHDE’s selectivity and interaction mechanisms, progressing from simplified systems toward increasingly complex membrane models. Trypanosomatidae parasites, including and , possess membranes enriched with unsaturated lipids such as phosphatidylcholine (PC) and phosphatidylethanolamine (PE), which contribute to membrane compressibility and structural adaptability.^17–19^ We previously used saturated lipids, such as dipalmitoylphosphatidylcholine (DPPC),^11^ since they provide a stable and well-characterized model system to study compound–lipid interactions under controlled conditions. Saturated lipids form highly ordered and condensed membranes with minimal compressibility at room temperature, ensuring reproducible experimental conditions and consistent interpretation of results. Their simple and predictable behavior serves as a reference point for comparison with more complex systems, such as unsaturated or mixed lipid membranes, allowing researchers to understand how the lipid structure influences compound interactions. Furthermore, saturated lipids like DPPC are relevant to certain mammalian membranes, such as lung surfactants, and their well-defined phase behavior offers insights into how compounds affect lipid surface arrangement, compressibility, and phase transitions. This foundational approach is essential for systematically evaluating the interactions of MDHDE before extending the analysis to more dynamic unsaturated lipid systems, providing a comprehensive understanding of compound selectivity and membrane targeting.

In this work, we expanded the knowledge on the interaction of MDHDE with models of membranes going to other characteristics of compressibility: 1,2-dioleoylphosphatidylcholine (DOPC) and 1,2-dioleoylphosphatidylethanolamine (DOPE), both unsaturated lipids, were employed. In contrast, cholesterol, a defining component of condensed mammalian membranes, was included to simulate the mammalian plasma membrane environment.^20,21^ The plasma membrane of protozoan parasites is increasingly recognized as a critical target for antiparasitic drugs, and some surface pressures in monolayers can be selected to mimic the thermodynamic properties of bilayers.^22,23^ Additionally, these membranes exhibit dynamic changes in lipid composition and metabolism as the parasite adapts to different host environments.^21^

We have to reinforce that dioleoyl (DO) lipids were selected because their unsaturated hydrocarbon chains more accurately reflect the fluid nature of biological membranes compared to fully saturated lipids. While simplified, such monolayer models are widely accepted in membrane biophysics as useful tools for probing specific interactions and physicochemical behaviors of individual lipid components, especially when aiming to isolate the influence of specific features such as the headgroup identity or degree of saturation.^24–30^ This approach allows us to draw comparative insights into how MDHDE interacts with different lipid environments. Also, these lipids are amply employed in Langmuir monolayer studies for this purpose.^24–30^ Regarding cholesterol, it plays a key role in modulating membrane properties such as rigidity, permeability, and domain formation. Its inclusion, even as a single-component monolayer, is supported by several studies^31–34^ to understand its individual contributions to membrane behavior. Moreover, cholesterol is a fundamental component of lipid rafts and is especially relevant when exploring how bioactive molecules interact with ordered membrane domains.

Investigating the interaction of antiprotozoal compounds with such lipid models not only elucidates their mechanisms of action but also provides valuable insights into their selectivity and therapeutic potential. This approach highlights the importance of lipid interfaces in guiding the rational design of novel anti- agents.

## 2 Materials and Methods

### 2.1 General

DOPC, DOPE, and cholesterol were purchased from Sigma-Aldrich (St. Louis, MO, USA) and dissolved in CHCl_3_ (Synth, Diadema, Brazil) to prepare stock solutions at a concentration of 0.5 mg/mL (Figure 1). The subphase water used in the Langmuir monolayer experiments was purified using a Milli-Q-Plus system, achieving a resistivity of 18.2 MΩ cm, pH 5.5 due to carbon dioxide dissolution, and a surface tension of 72.8 mN/m at 20 °C. For structural characterization, ^1^H and ^13^C NMR spectra were recorded at 300 and 75 MHz, respectively, using a Bruker Ultrashield spectrometer (Bruker-Biospin, Germany). CDCl_3_ (Aldrich) was employed as the solvent, containing 1% TMS (tetramethylsilane) as the internal standard. Chemical shifts (δ) are reported in ppm, and coupling constants (*J*) in Hz. Chromatographic separations were performed on silica gel (Merck, 70–230 mesh), while analytical thin-layer chromatography (TLC, 0.25 mm thickness) utilized silica gel 60 PF_2__5__4_ (Merck).

**Figure 1:**
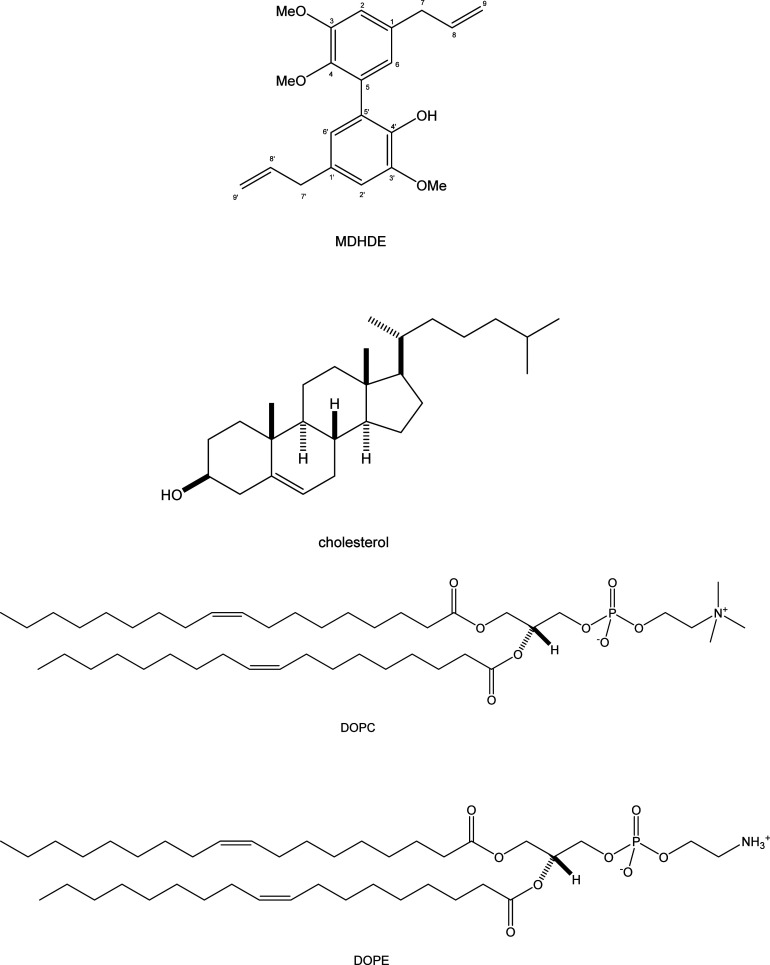
Structures of MDHDE, cholesterol, DOPC, and DOPE.

### 2.2 Preparation of MDHDE

Methyl dehydrodieugenol (MDHDE) was prepared by oxidative coupling from eugenol followed by methylation using CH_3_I, as previously reported.^11^ Briefly, from 30 mg of eugenol was obtained 7 mg of MDHDE.

#### 2.2.1 Methyl Dehydrodieugenol (MDHDE)

Amorphous solid. ^1^H NMR (CDCl_3_, 300 MHz): δ 6.36 (s, H-2/H-2′), 6.67 (s, H-6/H-6′), 3.31 (d, *J* = 6.8 Hz, H-7/H-7′), 5.92 (m, H-8/H-8′), 5.03 (m, H-9/H-9′), 3.84 (s, 3-OMe), 3.83 (s, 3′-OMe), 3.58 (s, 4-OMe). ^13^C NMR (CDCl_3_, 75 MHz): δ 125.5 (C-1), 131.2 (C-1′), 123.0 (C-2), 123.4 (C-2′), 141.4 (C-3), 144.2 (C-3′), 148.0 (C-4), 152.6 (C-4′), 132.0 (C-5), 136.4 (C-5′), 137.2 (C-6), 137.7 (C-6′), 39.9 (C-7), 40.1 (C-7′), 111.0 (C-8), 112.0 (C-8′), 115.6 (C-9), 116.1 (C- 9′), 56.0 (3-OMe), 56.1 (3′-OMe), 61.2 (4-OMe).

### 2.3 Langmuir Monolayer Preparation

A Langmuir trough (mini, KSV Instruments) was employed to assemble the monolayers, using the Wilhelmy plate method to measure surface pressure. Langmuir monolayers were prepared by spreading a lipid solution in chloroform (CHCl_3_, 0.5 mg/mL) onto the air–water interface. The subphase consisted of water purified with a Milli-Q Plus system (resistivity, 18.2 MΩ cm; surface tension, 72.9 mN/m at 20 °C), and all experiments were conducted at room temperature (25 ± 1 °C). Mixed monolayers were created by cospreading the lipid and drug from CHCl_3_ solutions (7–10% mol drug). While other drug concentrations were tested, this proportion was chosen as it represented the limit at which the compound exhibited measurable effects on the monolayer (e.g., expansion or condensation). Moreover, the low drug concentration reflects physiological conditions, where interactions with membranes occur at minimal levels.

As DO lipids are susceptible to oxidation, all lipid solutions were freshly prepared on the day of each experiment, and measurements were conducted within a maximum of 2 h after preparation. This precaution significantly minimizes oxidative degradation and ensures reproducibility and reliability of the data.

Surface pressure–area (π–*A*) and surface potential–area (Δ*V*–*A*) isotherms were recorded after a 20 min waiting period for chloroform evaporation. The monolayers were compressed at a rate of 10 Å^2^ molecule^–1^ min^–1^ until 30 mN/m to avoid collapse. To add MDHDE, the monolayers were expanded, MDHDE was incorporated, 30 min was waited for homogenization, and the monolayer was recompressed. Surface potential values were measured using a Kelvin probe, modeling the monolayer as a three-layer capacitor with varying dielectric constants, based on the Demchak–Fort approach.

For dilatational rheology measurements, monolayers were compressed to a surface pressure of 30 mN/m, followed by a 30 min stabilization period during which the barriers were adjusted as needed to maintain constant pressure. Subsequently, the monolayer underwent 20 cycles of compression and expansion with a 1% area variation at a frequency of 20 mHz. The complex dilatational surface modulus (*E*) was calculated using the equation *E* = −*A*(Δπ/Δ*A*)_T_, with values averaged over all cycles. The phase shift (φ) between surface pressure and area oscillation was used to determine the elastic (*E*′) and viscous (*E*″) components of the modulus, calculated as *E*′ = *E* sin φ and *E*″ = *E* cos φ, respectively. These experiments provided insights into the monolayer’s mechanical behavior, including its dilatational elasticity and surface viscosity.

Polarization-modulation infrared reflection-absorption spectroscopy (PM-IRRAS) was performed using a KSV PMI 550 instrument (KSV Instruments, Ltd., Helsinki, Finland) at a fixed incidence angle of 80°. The monolayers were compressed to a surface pressure of 30 mN/m, selected as it corresponds to the lateral pressure typically observed in natural membranes.^22,23^ Surface pressure was maintained at 30 mN/m by dynamic barrier adjustments during spectrum acquisition, with the same volume proportions used in the surface pressure–area isotherms.

Interfacial morphology was analyzed using a mini-BAM microscope (KSV-Nima) after compressing the monolayers to 30 mN/m with the same volume proportions used in the surface pressure–area isotherms. Rheological properties were assessed using the oscillating barrier method, where the monolayers were compressed to 30 mN/m, and the film area oscillated by 2% for 10 cycles of compression and decompression at a frequency of 20 mHz. Each experiment was repeated at least three times to ensure reproducibility.

## 3 Results

First, we have to comment that for all lipids used in this study, no significant differences were observed between the first, second, or third compression isotherms when going to 30 mN/m and expanded to the maximum area, indicating that there is no appreciable loss of the insoluble lipid material to the aqueous subphase. Therefore, any shifts observed in the isotherms upon addition of MDHDE cannot be attributed to lipid rearrangement during prior compression and expansion.

Figure 2 displays the surface pressure–area (π–*A*) isotherms for all the lipids used in this study. The isotherms of DOPC and DOPE exhibit characteristics indicative of less elastic monolayers compared to cholesterol.^35–37^ Specifically, the surface pressure for DOPC and DOPE increases more gradually with compression, reflecting the compressible nature of these unsaturated lipids. In contrast, cholesterol forms a more condensed and rigid monolayer, as evidenced by the steeper increase in surface pressure upon compression.

**Figure 2:**
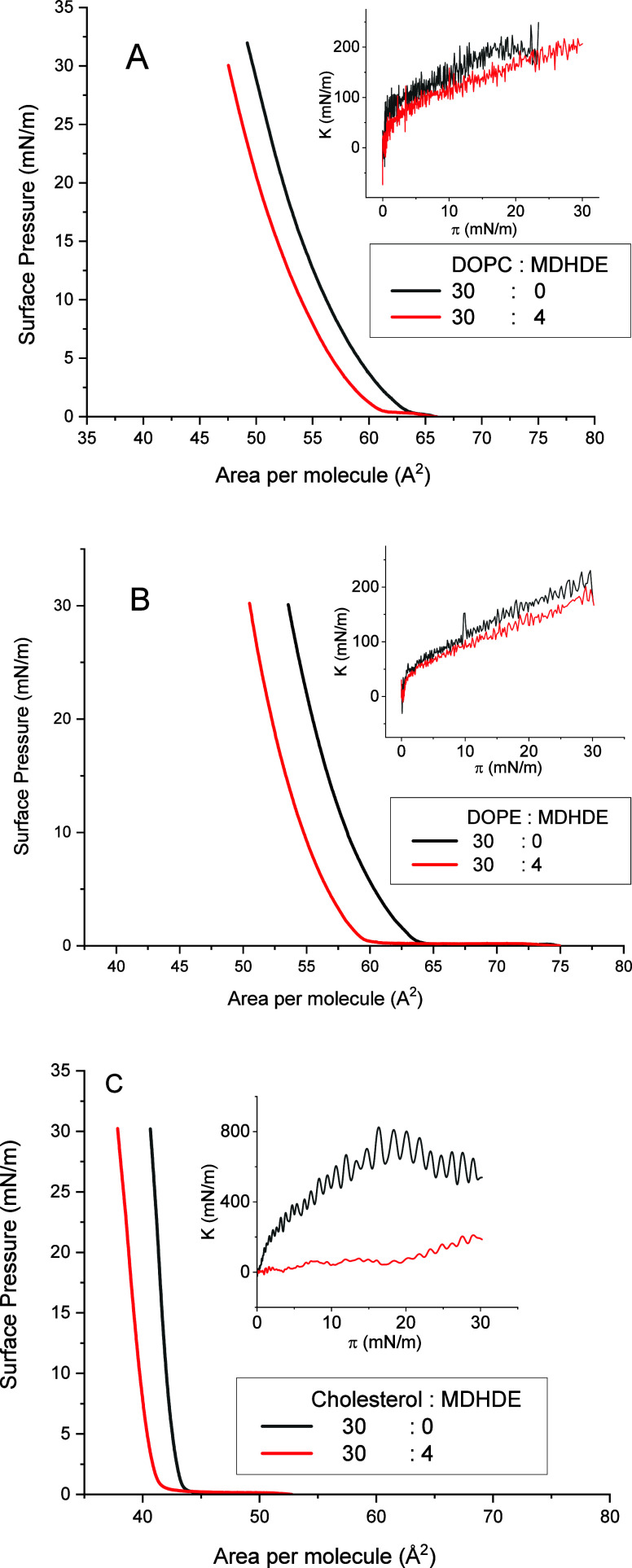
(A–C) Surface pressure–area (π–*A*) isotherms for the lipid monolayers without or with MDHDE (volume proportions indicated in the legend). Insets show the surface compressional modulus (*K*) extracted from the π–*A* isotherms.

Various MDHDE concentrations were tested, and the drug-to-lipid ratio presented here represents the limit at which the effect remains consistent. At higher concentrations, the effects became irregular, likely due the fact that at higher surface concentrations, MDHDE likely exceeds its miscibility limit with the lipid monolayer, leading to phase separation or formation of multilayer aggregates, which disrupt the reproducibility and linearity of the isotherms. Data on a selected range of concentrations are shown in the Supporting Information (Figure S1). This type of behavior has been observed in a Langmuir study involving amphiphilic drugs and other surface-active compounds.^38^ Also, the range of concentrations was selected based on previously reported results for saturated lipids^11^ and maintained low to approximate physiological conditions. In all cases, incorporation of MDHDE resulted in a noticeable shift of the isotherms toward lower molecular areas.

The insets of Figure 2 present the surface compression modulus (*K* = −*A*(dπ/d*A*)), calculated using the Davies and Rideal approach,^39^ which reflects the mechanical properties of the monolayers. For DOPC and DOPE, maximum *K* values were in the range of 150–250 mN/m, consistent with a liquid-expanded state. In contrast, cholesterol exhibited significantly higher *K* values, reaching 600–700 mN/m, corresponding to a highly condensed and rigid monolayer.

Upon incorporation of MDHDE, the compressional modulus (*K*) decreased in all lipid systems. For cholesterol, the reduction was more pronounced than for DOPC and DOPE, and the effect was less significant.

Figure 3 illustrates the stability of the lipid monolayers compressed to a surface pressure of 30 mN/m, a value representative of the lateral pressure found in natural membranes.^22,23^ Monolayer instability is typically observed as a decay in surface pressure over time and may result from the inability of molecules at the interface to maintain equilibrium under compression.^40,41^ When the monolayer is compressed to a high surface pressure (e.g., 30 mN/m), above its thermodynamic equilibrium point, the system enters an overcompressed state. In this state, lateral molecular stress increases, and the film may undergo relaxation processes such as molecular rearrangement, partial collapse, or formation of aggregates or multilayers. These processes result in a gradual decrease in surface pressure as the monolayer relaxes toward a new equilibrium. However, upon complete expansion, the initial state of the monolayer should be re-established.

**Figure 3:**
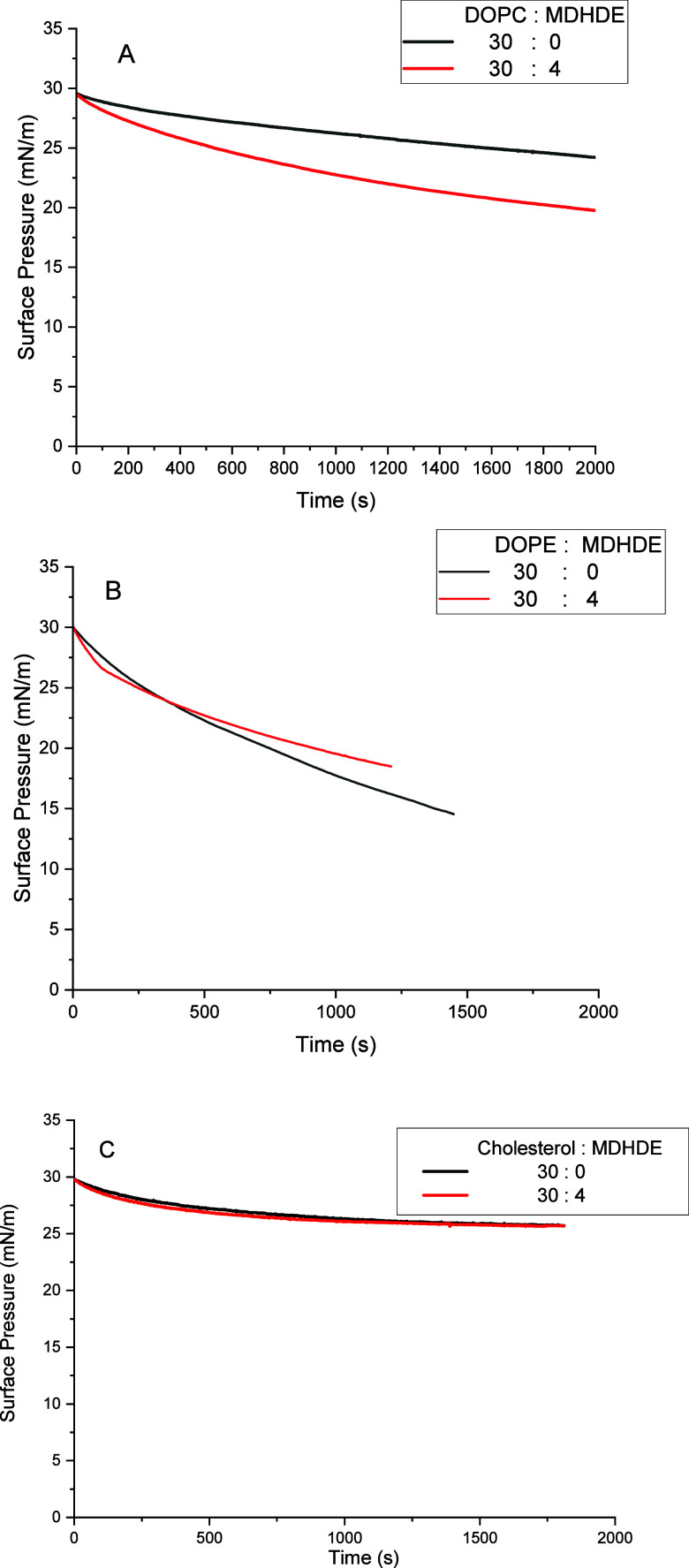
(A–C) Surface pressure–time isotherms for the lipid monolayers without or with MDHDE (volume proportions indicated in the legend) compressed to 30 mN/m and kept at a constant area.

For DOPC monolayers, the incorporation of MDHDE led to an increased surface pressure decay, indicating reduced stability. In contrast, for DOPE, MDHDE incorporation resulted in a lower decay in surface pressure, suggesting improved monolayer stability. For cholesterol, no significant difference in monolayer stability was observed with MDHDE incorporation.

The dilatational surface elasticity data presented in Table 1 show how the mechanical properties of lipid monolayers respond to the incorporation of MDHDE. For DOPC, the complex dilatational modulus (E*) increased significantly from 17.40 to 32.44 mN/m. Both the elastic (*E*′) and viscous (*E*″) components increased proportionally, while the phase angle (φ) remained stable at approximately 0.70, indicating that the relative balance between elastic and viscous behavior was maintained despite the enhanced rigidity.

**Table 1:** Dilatational Surface Elasticity Values for the Lipid Monolayers, with and without MDHDE^a^

	E* (mN/m)	*E*’ (mN/m)	*E*’’ (mN/m)	θ (rad)
DOPC	17.40	13.52	10.95	0.68
DOPC + MDHDE	32.44	24.74	20.94	0.70
DOPE	67.21	64.57	18.64	0.28
DOPE + MDHDE	65.19	62.77	17.47	0.27
cholesterol	249.92	247.77	32.77	0.13
cholesterol + MDHDE	213.01	210.19	34.56	0.6

a At the same volume proportions used in the isotherms. Each value represents the average of five measurements, with a maximum error margin of ±8%.

It is important to stick out the contrast between the effects observed on the compressional modulus and the dynamic elasticity. The slight decrease in the compressional modulus observed in the surface pressure–area isotherms of DOPC monolayers upon MDHDE incorporation suggests a modest reduction in monolayer rigidity under quasi-static compression. This parameter primarily reflects the monolayer’s thermodynamic response to compression at quasi-equilibrium conditions. In contrast, the observed increase in dynamic surface elasticity after relaxation and oscillatory cycles indicates enhanced viscoelastic behavior under time-dependent mechanical perturbation. This apparent contradiction highlights the fact that static and dynamic measurements probe different aspects of monolayer mechanics: while the isotherm-derived compressional modulus captures equilibrium compressibility, the dilatational modulus reflects the film’s ability to resist area changes over time, including potential restructuring, relaxation, and interfacial reinforcement effects. MDHDE may induce subtle reorganization or tighter headgroup interactions during relaxation, leading to a stiffer response under dynamic conditions despite a slight decrease in the modulus at equilibrium.

In the case of DOPE, E* showed only a slight decrease from 67.21 to 65.19 mN/m upon MDHDE addition. The elastic and viscous moduli were only marginally affected, and the phase angle remained low (around 0.27–0.28), reflecting a dominantly elastic behavior that persisted with MDHDE incorporation.

For cholesterol, MDHDE caused a decrease in E* from 249.92 to 213.01 mN/m. While the elastic modulus decreased slightly, the viscous component increased from 32.77 to 34.56 mN/m. This shift was accompanied by a pronounced increase in the phase angle from 0.13 to 0.60, indicating a transition toward a more dissipative mechanical response.

Figure 4 presents the surface potential–area isotherms for DOPC, DOPE, and cholesterol monolayers, with and without MDHDE. These measurements reflect the arrangement and orientation of dipolar groups at the interface during compression.

**Figure 4:**
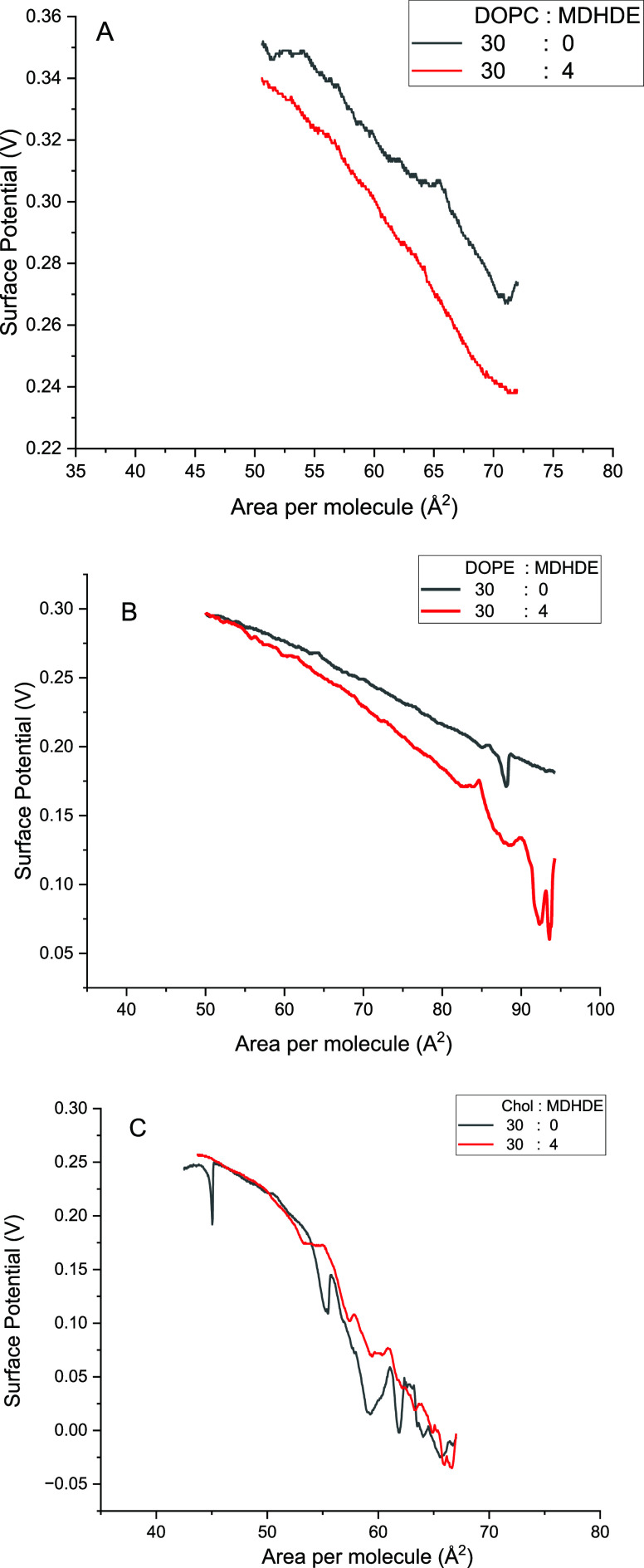
(A–C) Surface potential–area (Δ*V*–A) isotherms for the lipid monolayers without or with MDHDE (volume proportions indicated in the legend).

For DOPC, the incorporation of MDHDE caused a marked and sustained decrease in surface potential throughout the compression process until collapse.

For DOPE, a decrease in surface potential was observed at the beginning of compression, but the potential values for MDHDE-containing and pure monolayers converged at higher surface pressures. For cholesterol, no significant change in surface potential was observed upon MDHDE incorporation during compression.

Figure 5 shows Brewster angle microscopy (BAM) images of DOPC, DOPE, and cholesterol monolayers, both in the absence and presence of MDHDE, recorded after compression to a surface pressure near 30 mN/m. BAM provides visual insight into monolayer morphology, including homogeneity, domain formation, and molecular surface density.

**Figure 5:**
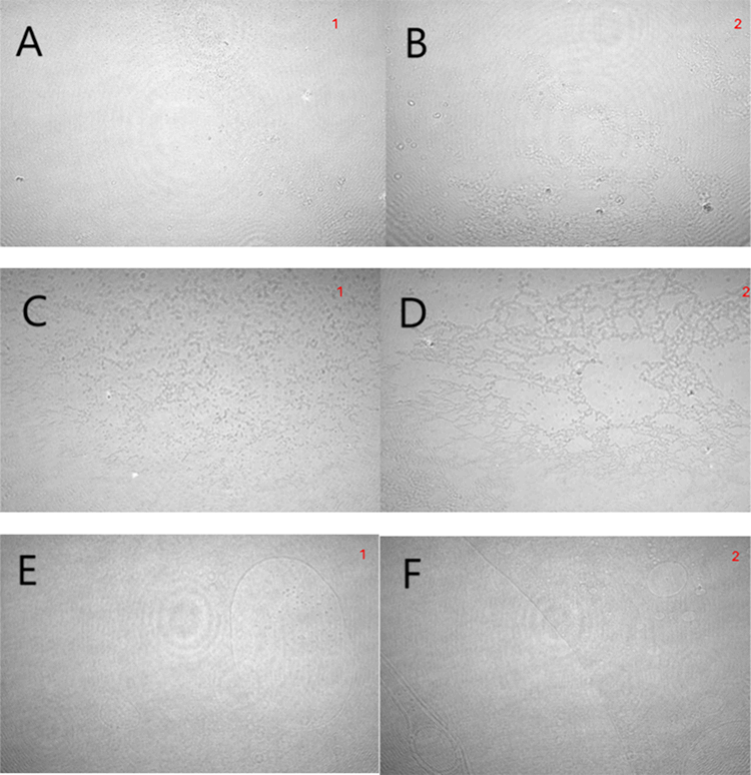
BAM images for the lipid monolayers without or with MDHDE (volume proportions indicated in the legend). (A, C, and E) pure lipids (DOPC, DOPE, and cholesterol, respectively) and (B, D, and F) with MDHDE (DOPC, DOPE, and cholesterol, respectively). Volume proportions: 30:4. Scale: 3600 × 2400 μm.

Under the experimental conditions, DOPC and DOPE remain in the liquid-expanded (LE) state, where classical phase separation is not typically expected for single-component systems. Nonetheless, the BAM images revealed optical anisotropies—interpreted as lateral heterogeneities rather than phase coexistence. These contrasts likely result from local differences in molecular orientation, molecular density, or thickness variations, particularly under high compression, and are consistent with structural rearrangements reported in the literature for pure lipids.^42,43^

For DOPC, BAM revealed small, scattered domains, which increased in number and density upon MDHDE incorporation, indicating enhanced heterogeneity in the monolayer. For DOPE, the BAM images showed dark domains characteristic of dense molecular regions. With MDHDE incorporation, these domains grew larger and fused into continuous regions, suggesting a change in domain behavior.

For cholesterol, BAM images showed a uniform morphology with no significant domain formation, and this pattern remained unchanged in the presence of MDHDE.

It is important to emphasize that even in monolayers composed of a single lipid in the LE phase, local differences in molecular orientation, molecular density, or thickness variations, especially at a higher surface pressure (e.g., ∼30 mN/m), can generate sufficient contrast to be detected by BAM. These contrasts may result from subtle structural rearrangements or transient ordering phenomena, which are known to occur even in homogeneous systems and have been reported in the literature.^41,42^ Thus, the features seen in our BAM images reflect real morphological changes and not imaging artifacts and reveal including effects from polar heads.

We have to emphasize that the heterogeneities observed are not artifacts, but rather real morphological features, likely reflecting local variations in molecular packing, orientation, or thickness, particularly at high surface pressures. While classical phase separation is not expected for single-component monolayers in the liquid-expanded state, the literature shows that optical anisotropies can emerge from subtle structural rearrangements under compression (e.g., transient clustering, orientational defects, or early domain formation), especially when a bioactive compound is introduced. We have expanded the discussion to better contextualize these features and provided a relevant reference,^44^ emphasizing that these morphological changes likely result from MDHDE-induced perturbations, rather than imaging artifacts.

Figure 6 presents the PM-IRRAS spectra of DOPC, DOPE, and cholesterol monolayers compressed to a surface pressure of 30 mN/m, providing insight into the molecular-level effects of MDHDE on a lipid monolayer. The spectra are divided into two key regions: the hydrophobic region (2800–3000 cm^–1^), which reflects the conformation of lipid acyl chains through CH_2_ stretching vibrations,^45^ and the hydrophilic region (1000–1700 cm^–1^), which includes vibrations related to headgroup and water interactions.

**Figure 6:**
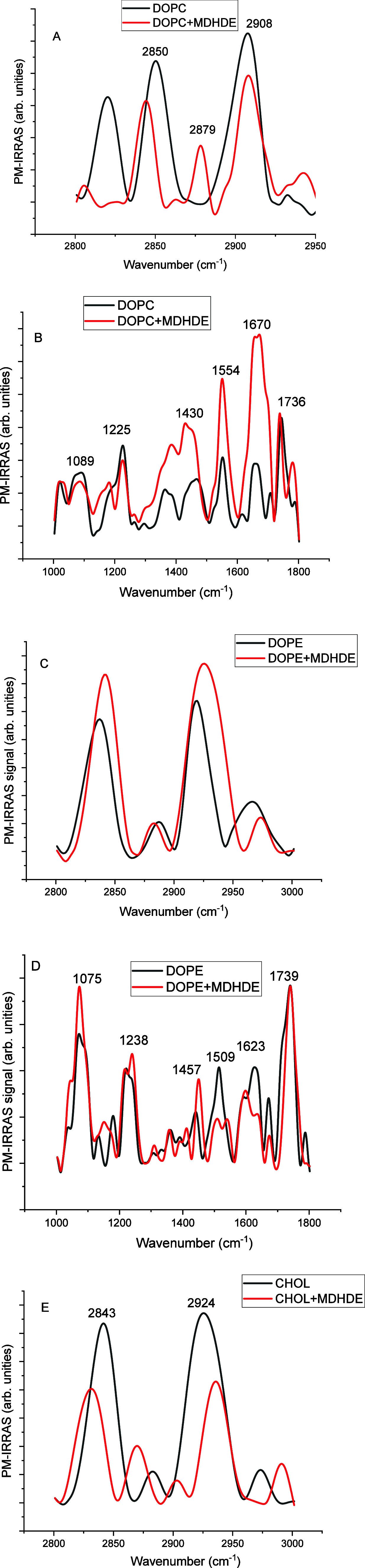
(A–E) PM-IRRAS spectra for the lipid monolayers without or with MDHDE at 30 mN/m (volume proportions: 30:4).

In the hydrophobic region, the characteristic symmetric (∼2850 cm^–1^) and asymmetric (∼2910–2920 cm^–1^) CH_2_ stretching bands exhibited distinct responses upon MDHDE incorporation. For DOPC and cholesterol monolayers, these bands shifted to lower wavenumbers, indicating an increased proportion of all-trans conformers. In contrast, DOPE showed a shift of the same bands toward higher wavenumbers following MDHDE addition, suggesting a decrease in trans conformers.

In the hydrophilic region, the spectra of the phospholipid monolayers displayed clear changes upon MDHDE incorporation. Shifts in the phosphate stretching (1000–1300 cm^–1^) and ester carbonyl bands (above 1700 cm^–1^) were observed, along with variations in the intensity of CH_2_ angular bending vibrations around 1450 cm^–1^. These changes suggest alterations in the polar headgroup environment, including modifications in intermolecular interactions or conformational adjustments between headgroups, which could influence their effective molecular area. In fact, bioactive compounds, such as MDHDE, may alter the hydration, hydrogen bonding, or orientation of polar headgroups.^46–50^ Additionally, in the region between 1500 and 1700 cm^–1^, associated with water molecules interacting with lipid headgroups, MDHDE caused noticeable shifts and changes in band intensity, pointing to altered hydration dynamics in the monolayer interface.

Bioactive compounds interacting with lipid monolayers can induce significant changes in the conformation and dynamics of the polar headgroup region, particularly through alterations in hydration, hydrogen bonding, and molecular orientation. Such effects have been reported for antimicrobial peptides, alkaloids, and amphiphilic drugs, which may disrupt the interfacial water structure or form specific hydrogen bonds with lipid headgroups, thus modifying the monolayer properties.^51^ These interactions can lead to reorientation or tilting of the headgroups, affecting dipole potential and surface packing.^52^ Moreover, compounds capable of forming hydrogen bonds or displacing water molecules at the interface may alter lipid phase behavior and membrane elasticity, with implications for membrane permeability and signaling pathways.^53^ Therefore, even in the absence of deep insertion into the hydrophobic core, subtle interactions at the headgroup level can result in pronounced biophysical effects.

For cholesterol, which lacks bulky polar headgroups, no distinct bands appeared in the hydrophilic region, as expected. However, changes were still detected in the hydrophobic region, where the CH_2_ stretching bands shifted to lower frequencies upon MDHDE addition, suggesting increased methylene all-trans conformers in the more rigid and tightly packed lipid system.

Taken together, the PM-IRRAS results demonstrate that MDHDE interacts with the lipid monolayers in both hydrophobic and hydrophilic regions, with distinct effects depending on the lipid type and structural features.

Table 2 summarizes the comprehensive set of results obtained from the various experimental techniques employed—π–*A* isotherms, compression modulus measurements, surface potential analysis, Brewster angle microscopy (BAM), and PM-IRRAS—allowing for a comparative evaluation of MDHDE’s interactions with different lipid monolayers. This integrative model highlights how MDHDE modulates the physical and structural properties of DOPC, DOPE, and cholesterol monolayers in a lipid-dependent manner.

**Table 2:** Effect of Methyl Dehydrodieugenol (MDHDE) on Each Lipid Monolayer Based on the Physicochemical Characterization

	effect on DOPC	effect on DOPE	effect on cholesterol
π–*A* isotherms	condensation	condensation	condensation
surface elasticity	slightly more compressible	slightly more compressible	significantly more compressible
dynamic elasticity	increases elasticity and keeps viscosity	keeps elasticity and keeps viscosity	decreases elasticity and increases viscosity
tensiometric stability	decreases more π	decreases less π	keeps the level of π decay
surface potential	decreases	decreases	keeps
BAM	changed morphology	changed morphology	unchanged morphology
PM-IRRAS all-trans conformer ratio	increases	decreases	increases

## 4 Discussion

The data collectively show that MDHDE interacts with lipid monolayers in a lipid-specific manner, modulating structure, elasticity, and interfacial properties through distinct mechanisms. π–*A* isotherms revealed that DOPC and DOPE form more compressible, loosely condensed monolayers compared to cholesterol. Their gradual pressure increase under compression reflects the influence of unsaturated acyl chains that hinder more condensed states, while cholesterol exhibited a steeper, more rigid response due to its condensing effect.

Upon MDHDE incorporation, isotherms for all lipids shifted to lower molecular areas, suggesting tighter surface density or changes in the molecular orientation at the air–water interface. This effect was more pronounced for the unsaturated lipids, particularly DOPE, indicating enhanced susceptibility to MDHDE insertion. Compression modulus data supported this interpretation, as all systems exhibited reduced rigidity after MDHDE addition, with the greatest drop observed in DOPE, reflecting its higher affinity for MDHDE. In contrast, DOPC showed a moderate response, while cholesterol, though initially rigid, experienced a notable decrease in modulus, suggesting a smoother response to the compression as the compressibility altered.

The dilatational results highlight the lipid-specific effects of MDHDE on monolayer viscoelasticity. In DOPC, the marked increase in both elasticity and viscosity suggests that MDHDE intercalates into the loosely packed unsaturated chains, enhancing molecular cohesion and increasing resistance to deformation. The stable phase angle implies that MDHDE amplifies the mechanical response without altering the overall balance between elastic and viscous properties.

For DOPE, the minor decrease in E* and the preservation of a low phase angle indicate that MDHDE does not disrupt the monolayer significantly but may instead contribute to its stability. This behavior could stem from favorable hydrogen bonding interactions between the ethanolamine headgroups of DOPE and the polar moieties of MDHDE, leading to a cohesive, yet slightly more compressible, monolayer.

In cholesterol monolayers, MDHDE exerts a softening effect by reducing the elastic modulus while increasing the viscous component and phase angle. This suggests that although cholesterol-rich films are highly rigid, MDHDE is able to partially alter the molecular arrangement of the sterol rings through hydrophobic interactions, resulting in a more dissipative and less elastic film.

Collectively, these observations reinforce MDHDE’s role as a selective modulator of membrane mechanics. Its pronounced effect on DOPC and mild stabilization of DOPE, coupled with limited yet detectable influence on cholesterol, reflect its potential to differentially affect biological membranes based on lipid composition, particularly those rich in unsaturated phospholipids like those of protozoal parasites.

These mechanical effects were echoed in stability measurements. MDHDE destabilized DOPC monolayers, increasing surface pressure decay, whereas it enhanced DOPE stability, likely through specific interactions between the ethanolamine headgroups and the MDHDE hydroxyl moiety. Cholesterol remained largely unaffected, reinforcing its structural resilience.

Surface potential data further clarified these interactions. In DOPC, MDHDE caused a persistent reduction in surface potential, pointing to alteration in the molecular environment in the headgroup region. For DOPE, a transient decrease was observed, followed by recovery under compression, suggesting molecular rearrangement and stabilization. Cholesterol’s surface potential remained unchanged, indicating negligible perturbation of its interfacial electrostatics.

BAM imaging reinforced these observations. DOPC showed increased domain density with MDHDE, consistent with greater lateral heterogeneity. DOPE exhibited domain coalescence, reflecting enhanced molecular ordering. Cholesterol retained a homogeneous morphology, confirming its resistance to disruption.

PM-IRRAS spectra revealed region-specific effects of MDHDE. In the hydrophobic region, modulation of chain dynamics can occur with the introductions of drugs. DOPC and cholesterol showed a shift to lower wavenumbers, indicative of a higher all-trans/gauche ratio, representing a shift in acyl chain conformations toward trans rotamers, while DOPE shifted toward higher frequencies, reflecting decreased all-trans conformers. These contrasting effects suggest that MDHDE both promotes acyl chain conformation changes, depending on the lipid context. In the hydrophilic region, MDHDE altered phosphate, carbonyl, and water-related bands in DOPC and DOPE, revealing its influence on headgroup arrangement and hydration dynamics. As expected, cholesterol showed no such features due to its nonpolar headgroup.

It is important to emphasize that not all cases of monolayer condensation are necessarily associated with an increase in trans conformers. While the shift of π–*A* isotherms toward lower molecular areas is commonly interpreted as increased molecular density, this can result from specific interactions at the polar headgroup level. In the case of DOPE, MDHDE may promote condensation through lateral interactions, such as hydrogen bonding with ethanolamine groups, or by stabilizing structural defects, thereby facilitating molecular approaching and reducing lateral repulsions. This condensation can occur even as the acyl chains adopt more gauche conformers, reflecting local disorder. In such scenarios, increased surface density reflects stronger attraction between headgroups, while the hydrophobic tails retain flexibility to accommodate the new arrangement. This may involve horizontal molecular displacement, lateral drug–lipid interactions, or the formation of precollapsed domains, as indicated by the PM-IRRAS data. This decoupling of hydrophilic and hydrophobic ordering is consistent with literature reports where amphiphilic compounds induce tighter headgroup approaching while simultaneously increasing chain fluidity.^23,54^

Cholesterol demonstrates a degree of resilience when considering the full set of experimental data. Despite exhibiting some changes in compressibility, such as a partial reduction in the compressional modulus and signs of acyl chain reorganization observed via PM-IRRAS, it largely retains its morphological and electrostatic stability, as evidenced by the unaltered BAM images and surface potential curves. This partial resistance to disruption suggests that the effects induced are better described as modulation rather than complete disorganization. Overall, cholesterol shows a limited yet measurable susceptibility to structural perturbation, while maintaining its inherently rigid and ordered character.

Taken together, these results outline a model where MDHDE intercalates into DOPC, affecting headgroup interactions, leading to condensation and morphological heterogeneity. In DOPE, MDHDE stabilizes the monolayer by interacting with ethanolamine headgroups, facilitating domain coalescence and preserving structural integrity. For cholesterol, MDHDE modestly condenses the monolayer via hydrophobic interactions but induces minimal disruption.

Comparison with saturated lipid systems from a previous study^11^ further highlights the role of lipid saturation and headgroup chemistry in governing MDHDE’s effects. Differently from its action on DOPC and DOPE, MDHDE expanded DPPC and DPPG monolayers, suggesting an affinity for disordered acyl chains. Its condensing effect on DOPE parallels its behavior in DPPE, where stronger headgroup interactions dominate. Minimal effects on cholesterol align with its behavior in DPPS, reinforcing cholesterol’s function as a structural stabilizer.

Altogether, these findings suggest that MDHDE selectively modulates lipid arrangement and dynamics, with potential implications for membrane integrity and function in biological systems. The strong interactions with PE-rich membranes, such as those in protozoa, support its proposed antiparasitic activity, while the limited disruption of cholesterol-rich domains suggests selectivity that could minimize effects on host membranes. This comprehensive lipid-dependent behavior highlights MDHDE’s potential as a membrane-targeting therapeutic and sets the stage for further molecular-level structural characterization studies using tools like molecular dynamics simulations or neutron reflectometry.

## 5 Conclusions

This study investigated the interaction of methyl dehydrodieugenol (MDHDE), a natural antiprotozoal compound, with model lipid monolayers that mimic key features of protozoal and mammalian membranes. Surface pressure isotherms, compression modulus, and PM-IRRAS analyses revealed lipid-dependent effects: MDHDE increased membrane compressibility and altered molecular arrangement in DOPC and cholesterol, while stabilizing DOPE, likely via hydrogen bonding with ethanolamine headgroups. Surface potential and BAM imaging confirmed these selective interactions, with altered electrostatics and distinct morphological changes. These results underscore MDHDE’s ability to modulate membrane properties in a composition-dependent manner, supporting its potential as a selective, membrane-targeting therapeutic. Further studies with complex models and advanced techniques are needed to deepen our understanding and guide rational drug development.

## Supplementary Material



## Notes

Surface pressure–area (π–*A*) isotherms for the lipid monolayers (PDF)
