# Supplementary material for: Selective Modulation of Lipid Langmuir Monolayers by Methyl Dehydrodieugenol: Insights into Its Interaction with Compressibility-Modulating Lipid Interfaces for Antiprotozoal Applications
Source: Langmuir. 2025 Jul 14;41(29):19582–92. doi: 10.1021/acs.langmuir.5c02535 (PMC12312162; doi:10.1021/acs.langmuir.5c02535)
Supplement: Supplementary file 1 [file la5c02535_si_001.pdf]

Supplementary Information

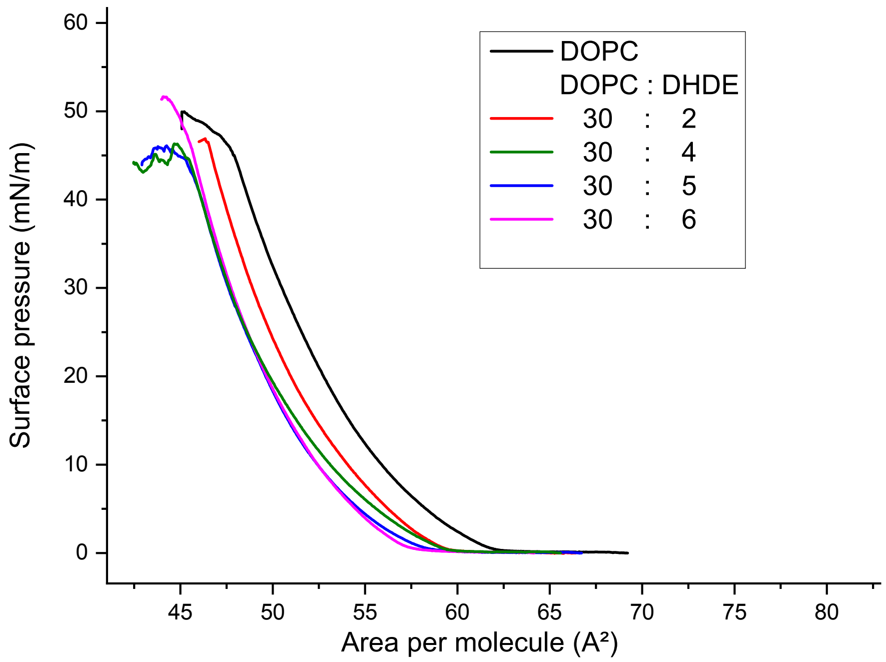

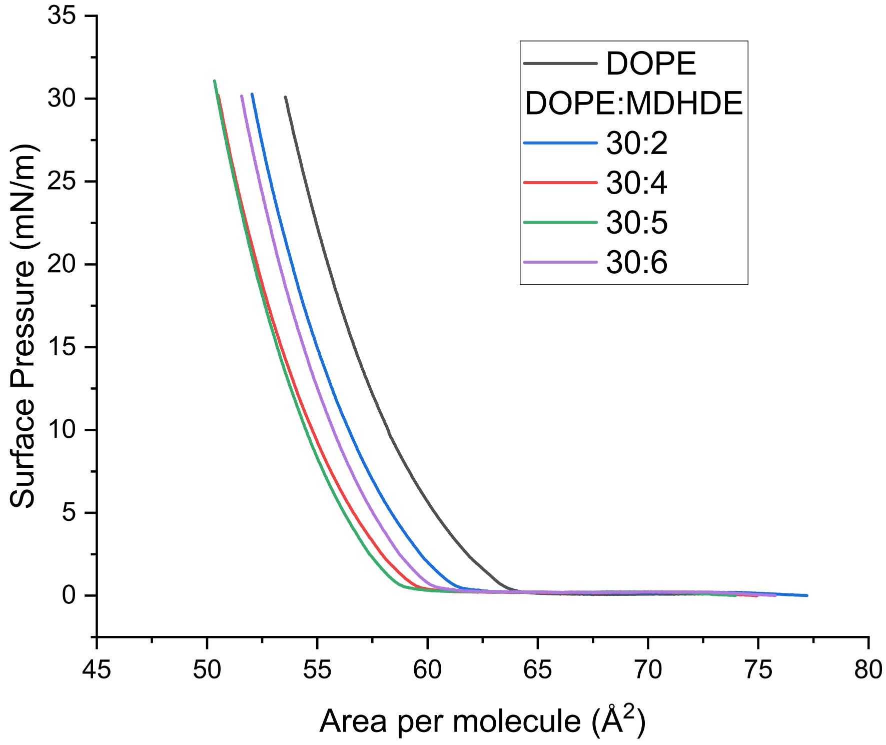

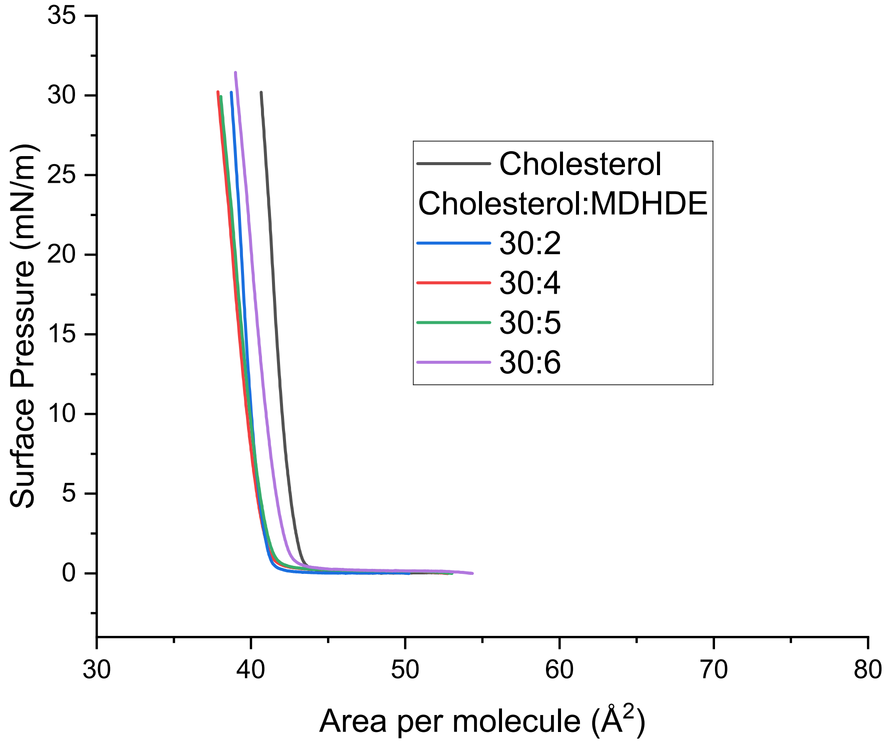

Figure S1: Surface Pressure-area (π-A) isotherms for the lipid monolayers without or with MDHDE (volume proportions indicated in the inset).
